# Correspondence

**Published:** 1878-08

**Authors:** Geo. L. Field

**Affiliations:** Local Committee American Dental Association


### CORRESPONDENCE.

To the Editor of the Register:—At the last meeting of the American Dental Association, which was held at Chicago, the following committee was appointed for local work: Drs. G. C. Daboll. Geo. L. Field and C. R. Butler. Unfortunately the two ends of the Committee have deemed it advisable to leave the country for a time, leaving the center only intact, and I being said center, have made the following arrangements for our next meeting at Niagara Falls. Grant's Hall has been secured for our meetings. Rates for delegates and their wives will be at the International Hotel three dollars per day. At the Cataract House, two dollars and a half. These include omnibus and baggage to and from the hotels. Rooms at either hotel may be engaged in advance. Those stopping at the International, are entitled to a free ticket of admission to Prospect Park during their stay. As to other places of interest at the Falls, I would say that no arrangements have been made, as yet for a reduction of rates for members of the Association, but I shall make it my business to see what concessions can be obtained. In railroad matters I have not succeeded in doing much. The New York and Erie will give their agents at Newark and Patterson instruction to sell excursion tickets to the Falls and return, good for the time of the Association, at sixteen dollars. Mr. Abbott also writes me that he will give reduced rates from other stations on the road. Those wishing to pass over this road would do well to correspond with John N. Abbott, General Passenger Agent, New York, at an early date. The Canada Southern and Great Western Roads of Canada, will sell tickets over their lines at half rates. The Toledo and Wabash Road will do the same.

In all cases the delegates will be required to present to the ticket agents a certificate from either the President or Secretary of the society he represents, certifying that he is a proper person to receive said reduction. These certificates can be obtained by the society officers, of the general ticket agents of the above mentioned roads. Michigan roads will make no reduction. If the Association met in that state half rates might be secured. I shall make no further effort in railroad matters, as it will be better for members living near one locality to make their own arrange ments and secure concessions for themselves. Again if I should be too successful in the committee work, I might be placed in a similar position again, and as this year will bring honors sufficient, I wish no more.

Respectfully,

GEO. L. FIELD,

Local Committee American Dental Association.

